# Supplementary material for: The Construction and Exploration of a Comprehensive MicroRNA Centered Regulatory Network in Foxtail Millet (Setaria italica L.)
Source: Front Plant Sci. 2022 May 6;13:848474. doi: 10.3389/fpls.2022.848474 (PMC9121102; doi:10.3389/fpls.2022.848474)
Supplement: Supplementary file 3 [file Data_Sheet_3.zip › Sit-miR395j_Seita.4G281500.1_2252_TPlot.pdf]

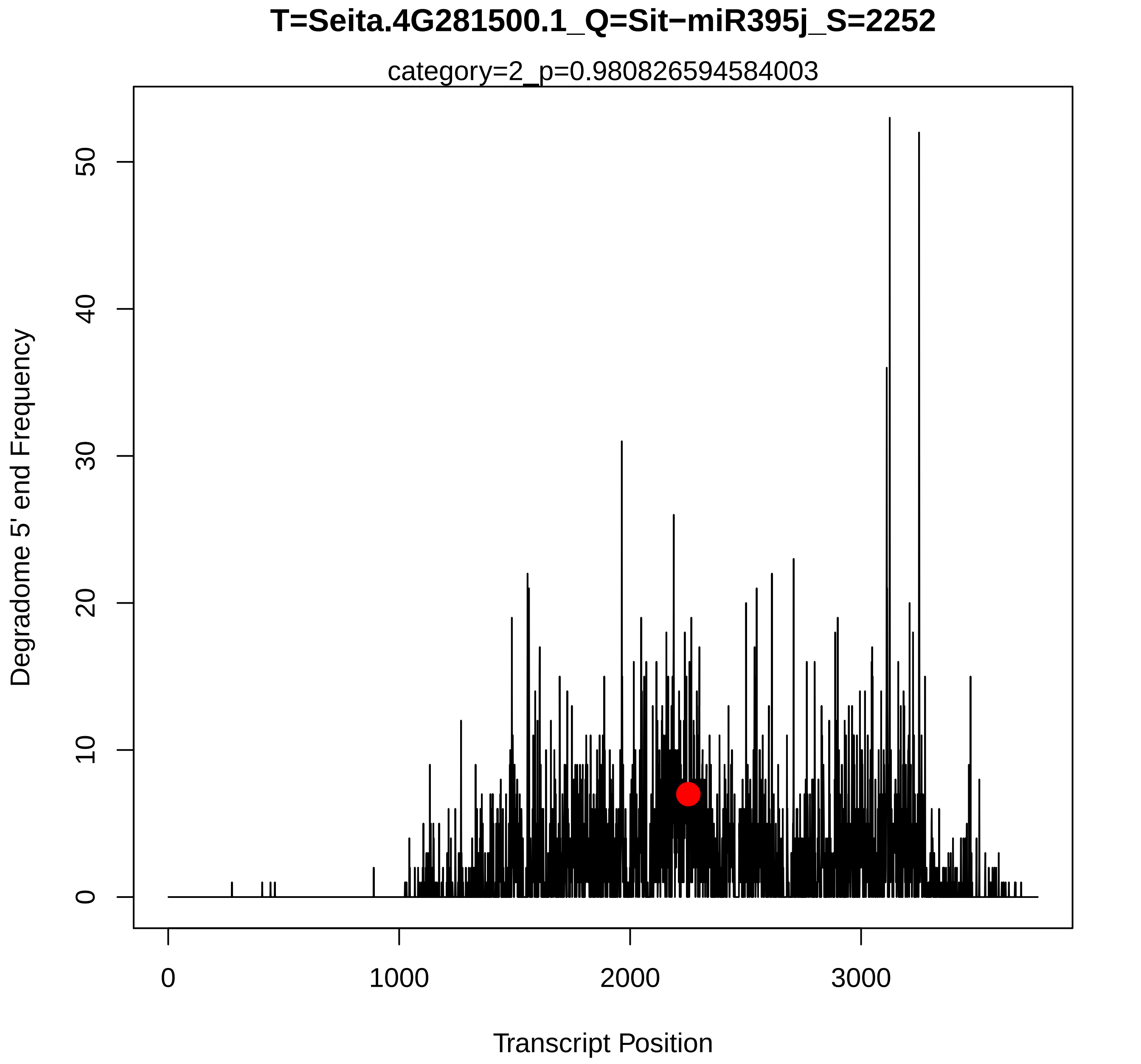
T=Seita.4G281500.1_Q=Sit−miR395j_S=2252
category=2_p=0.980826594584003
Degradome 5' end Frequency
0
10
20
30
40
50
0
1000
2000
3000
Transcript Position
